# Radiomics for the Detection and Prediction of Cancer Therapy-Related Cardiotoxicity

**DOI:** 10.1016/j.jacadv.2026.102942

**Published:** 2026-07-01

**Authors:** Abhinav Kandala, Amar Rai, Rahul Penumaka, Nicholas S. Wilcox, Benedicte Lefebvre, Paco E. Bravo, Michael G. Fradley

**Affiliations:** a Faculty of Medicine, University College London, London, United Kingdom; b Department of Internal Medicine, University of Pennsylvania, Philadelphia, Pennsylvania, USA; c Department of Surgery and Cancer, Imperial College London, London, United Kingdom; d Department of Medicine, Stanford University, Stanford, California, USA; e Division of Cardiology, Department of Medicine, Perelman School of Medicine, University of Pennsylvania, Philadelphia, Pennsylvania, USA; f Thalheimer Center for Cardio-Oncology, Abramson Cancer Center, Perelman School of Medicine, University of Pennsylvania, Philadelphia, Pennsylvania, USA; g Department of Radiology, Perelman School of Medicine, University of Pennsylvania, Philadelphia, Pennsylvania, USA; h Department of Medicine, Perelman School of Medicine, University of Pennsylvania, Philadelphia, Pennsylvania, USA

**Keywords:** cardio-oncology, cardiotoxicity, machine learning, prediction, radiomics

## Abstract

Modern cancer therapies have improved survival but unmasked the growing challenge of cancer therapy-related cardiotoxicity, a leading cause of morbidity and mortality in cancer survivors. Current surveillance strategies, relying on serial echocardiography and cardiac biomarkers, are reactive, detecting cardiac injury only after clinical symptoms emerge. Identifying fundamental, imaging-based biomarkers enables timely recognition of subclinical cardiotoxicity. Radiomics, the high-throughput extraction of quantitative features from routine medical images, provides a noninvasive method to characterize tissue pathophysiology beyond the limits of human visual perception. This review explores the emerging evidence of radiomics for the real-time detection and prediction of cardiotoxicity. We identify distinct radiomic signatures across imaging modalities and examine the challenge of personalized risk stratification utilizing radiomics, which show significant promise. This review concludes that although this novel field is currently limited by small studies lacking external validation, radiomics is poised to enable a paradigm shift in the field of cardio-oncology.

## Highlights

• Current functional surveillance is limited by variability and delayed detection.
• Radiomic signatures detect tissue heterogeneity preceding functional decline.
• Texture analysis via Echo, CT, and PET outperforms standard metrics.
• Translation requires large-scale validation of multimodal and multiomic models.

Modern cancer therapies have led to a substantial global decline (23.9%) in cancer mortality.^1^, ^2^, ^3^ However, improvements in therapeutic efficacy have paradoxically increased the burden of cancer therapy-related cardiotoxicity, a principal source of noncancer morbidity and mortality among cancer survivors. The clinical impact of cancer therapy-related cardiotoxicity is substantial, with an incidence of 26% in patients receiving chemotherapies and 10% to 30% 5 to 10 years following radiation therapy, with a mortality as high as 22.9 deaths per 100 patient-years.^4^, ^5^, ^6^, ^7^, ^8^ Cancer therapy-related cardiotoxicity manifests as a broad spectrum of cardiovascular insults, including myocardial dysfunction, heart failure, and coronary artery disease.^9^

Early detection significantly impacts survival and long-term cardiovascular health.^10^ For example, the transition from asymptomatic disease to symptomatic heart failure is associated with a decrease in 5-year survival from 96% to 75%.^11^ Current protocols are limited by detecting cardiotoxicity only after cardiac functional impairment, rather than at the subclinical stage. As per international guidelines, patients undergo baseline cardiovascular risk assessment, utilizing disease prediction models such as SCORE2 or SCORE2-OP, followed by serial imaging and cardiac biomarkers.^12^, ^13^, ^14^, ^15^, ^16^

The international guidelines for cardio-oncology surveillance have several limitations both in terms of imaging and biomarkers.^12^, ^13^, ^14^, ^15^, ^16^ Left ventricular ejection fraction (LVEF), the traditional gold standard of cardiac surveillance, is an insensitive, late-stage indicator of dysfunction. Its utility for early detection is further compromised by a measurement variability of over 10% with Biplane Simpson’s method, problematically mirroring the >10% decline often used to define cardiotoxicity (Table 1).^17^ Thus, making it difficult to reliably distinguish true subclinical change from simple measurement noise.^17^ Although more sensitive metrics like global longitudinal strain (GLS) have been introduced and shown to identify subclinical changes even with a normal LVEF, they are also hindered by measurement variability.^17^ GLS is subject to a measurement variability of 8.3% to 11% in addition to a lack of standardization across different imaging software and models, such as Tagging, CVI42, Segment, and Tomtec, further complicating its reproducibility.^18^

**Table 1:** Definition of Cardiotoxicity According to Different Guidelines

Organization, Year	Asymptomatic Cardiotoxicity	Symptomatic Cardiotoxicity	Guideline (Reference Number)
European Society of Cardiology (ESC), 2022	-	• Very Severe: HF requiring inotropic support, mechanical circulatory support, or consideration of transplantation	2022 ESC Guidelines on cardio-oncology developed in collaboration with the European Hematology Association (EHA), the European Society for Therapeutic Radiology and Oncology (ESTRO) and the International Cardio-Oncology Society (IC-OS)
	• Severe: new LVEF reduction to <40%	• Severe: HF hospitalization	
	• Moderate: new LVEF reduction by ≥10% to an LVEF of 40%-49% *or* new LVEF reduction by < 10 percentage points to an LVEF of 40%-49% and either new relative decline in GLS by >15% from baseline or new rise in cardiac biomarkers	• Moderate: need for outpatient intensification of diuretic and HF therapy	
	• Mild: LVEF ≥ 50% and new relative decline in GLS by >15% from baseline and/or new rise in cardiac biomarkers	• Mild: Mild HF symptoms, no intensification of therapy required	
European Society for Medical Oncology (ESMO), 2020	• LVEF decrease of ≥10% from baseline to >50% • GLS relative decrease of ≥12% from baseline, or an absolute decrease of ≥5%	• LVEF <40% or a ≥10% decrease to <50% accompanied by symptoms of HF • GLS relative decrease of ≥12% from baseline, or an absolute decrease of ≥5% and presence of clinical HF symptoms	Management of cardiac disease in cancer patients throughout oncological treatment: ESMO consensus recommendations
Canadian Cardiovascular Society (CCS), 2016	• >15% relative reduction in GLS from baseline • LVEF decrease of >10% from baseline to a value below 53% • Elevated troponin without symptoms • Elevated BNP levels without symptoms	• Development of clinical heart failure symptoms • LVEF decline >10% to below 50% • Elevated troponin or BNP associated with clinical symptoms	Canadian Cardiovascular Society Guidelines for Evaluation and Management of Cardiovascular Complications of Cancer Therapy
British Society of Echocardiography (BSE) and the British Society of Cardio-Oncology (BCOS), 2021	• Decline in LVEF >10% from baseline to a value <50% or a relative reduction in GLS >15% from baseline • No clinical symptoms	• Decline in LVEF >10% to <50%, with symptoms of heart failure OR GLS >15% decline, along with symptoms	BSE and BCOS Guideline for Transthoracic Echocardiographic Assessment of Adult Cancer Patients Receiving Anthracyclines and/or Trastuzumab
Sociedade Brasileira de Cardiologia (SBC), 2020	• Reduction in LVEF ≥10% from baseline, but still ≥50% • GLS reduction **≥** 15% relative to baseline	• LVEF <50% with clinical signs of heart failure • Signs of congestion or heart failure on physical exam	Diretriz Brasileira de Cardio-oncologia
American Society of Clinical Oncology (ASCO), 2021	• Decrease of ≥10% from baseline and final LVEF <55% • Reduction >11% from baseline • Troponin I or T elevated above assay-specific normal limit • BNP or NT-proBNP elevated	• LVEF <40% with clinical signs of heart failure • Troponin **>**0.07 ng/mL indicating ongoing myocardial injury • BNP >300 pg/mL	Management of Immune-Related Adverse Events in Patients Treated With Immune Checkpoint Inhibitor Therapy: ASCO Guideline Update

Definitions of asymptomatic and symptomatic cancer-therapy related cardiotoxicity with comparative analysis of clinical practice guidelines used as knowledge sources.

BNP = B-type natriuretic peptide; GLS = global longitudinal strain; HF = heart failure; LVEF = left ventricular ejection fraction; NT-proBNP = N-terminal pro–B-type natriuretic peptide.^12^, ^13^, ^14^, ^15^, ^16^

The multicenter SUCCOUR trial investigated GLS-guided vs LVEF-guided initiation of cardioprotective therapy and demonstrated no significant difference in LVEF at 3 years. Interpretation was limited by the low event rate, modest sample size, and predominance of early-stage breast cancer, reducing generalizability to higher-risk populations.^19^ In contrast, the SUCCOUR-MRI substudy found that among patients who developed an isolated GLS reduction after anthracycline therapy (but without an LVEF decline), initiation of cardioprotective therapy was associated with better preservation of magnetic resonance imaging (MRI)-measured LVEF at 12 months compared with usual care.^20^ However, the absolute magnitude of this LVEF benefit was modest, the durability beyond 12 months is unknown, and the use of MRI-LVEF, less common in clinical surveillance, limits applicability to real-world practice. In addition, myocardial tissue characterization did not demonstrate prevention of diffuse structural remodeling.^20^ Together, these highlight potential limitations of relying solely on GLS for subclinical detection of cancer therapy-related cardiotoxicity. Serial monitoring of troponin and natriuretic peptides (B-type natriuretic peptide and N-terminalpro–B-type natriuretic peptide) for early detection is also limited by poor predictivity and specificity.^21^^,^^22^ Evidence for their use remains inconsistent, with reported positive predictive values ranging from 0.44 to 0.57.^21^

Radiomics is an emerging field at the intersection of medical imaging and artificial intelligence (AI) that may address limitations of current modalities for detecting and predicting cardiotoxicity. It involves computationally extracting a vast array of quantitative features from medical images, translating pixel-level data into high-dimensional signatures that can quantify tissue heterogeneity, morphology, and texture, known as radiomic signatures.^23^ By identifying myocardial changes beyond the limits of human visual perception, radiomics offers the potential to detect and predict cardiotoxicity at a subclinical stage, potentially allowing for timely cardioprotective interventions to prevent clinical dysfunction.^24^

Rapid advancements in AI and machine learning have driven improvements in the application of radiomics across oncology, with several models combining radiomic features with clinical data, demonstrating clinical efficacy.^25^ Although its application in cardio-oncology is still emerging, it offers a significant opportunity to address an unmet clinical need. Few studies have explored how radiomics could be used to predict or detect cancer therapy-related cardiotoxicity, offering an opportunity to address an unmet need in the field.

Clinical relevance of radiomic findings requires an understanding of the pathophysiological substrate they are designed to detect, and crucially, how that substrate differs from the myocardial injury seen in more common cardiac diseases. Unlike the focal, territory-dependent infarction seen in ischemic heart disease, or the generalized global dysfunction typical of nonischemic-dilated cardiomyopathy, cancer therapy-related cardiotoxicity presents as a diffuse, progressive, and mechanistically unique pattern of myocardial injury. This toxicity is predominantly driven by overlapping pathophysiological processes. Cancer therapies have distinct cardiotoxicity mechanisms, with anthracyclines being frequently cited as the most common cardiotoxic agent. This drug inflicts direct oxidative injury through topoisomerase IIβ inhibition, reactive oxygen species generation, and mitochondrial dysfunction.^26^ This cellular cascade drives cardiomyocyte apoptosis and subsequent diffuse interstitial fibrosis, resulting in structural remodeling that emerges months before any measurable decline in LVEF.^26^ In addition, radiation therapy initiates a distinct trajectory of endothelial and vascular damage. Over a span of years to decades, this chronic pathway promotes sustained inflammatory and structural consequences, including pericarditis, accelerated coronary atherosclerosis, and pericoronary adipose inflammation.^27^ In particular HER2-targeted therapies such as trastuzumab act through mitochondrial HER2 signaling disruption without direct DNA damage, producing a potentially more reversible phenotype that differs texturally from anthracycline-related fibrosis.^26^ This distinction matters for radiomics because the spatial pattern of voxel heterogeneity, whether it reflects a focal scar, a diffuse fibrotic process, or adipose infiltration, could potentially generate a modality-specific radiomic signature that differs between these phenotypes.

This review evaluates the current landscape of radiomics for the detection and prediction of cancer therapy-related cardiotoxicity. We define detection as the identification of existing subclinical or established cardiotoxicity from imaging acquired during or after cancer therapy and prediction as the estimation of future cardiotoxicity risk from pretreatment or early-treatment imaging, before functional decline has occurred. We begin with an overview of the technical workflow of radiomics, followed by the application of radiomics across 3 principal imaging modalities, including positron emission tomography (PET)/computed tomography (CT), cardiac MRI (CMR), and echocardiography. In closing, we offer a forward-looking perspective on the key elements required to translate and integrate radiomics into patient care in cardio-oncology.

## Methodology

A literature review was conducted by an independent reviewer. We searched the Medline (PubMed), Embase, Scopus, and Central databases for all relevant articles, without publication date restrictions. This state-of-the-art review employed a formal search strategy and Preferred Reporting Items for Systematic Reviews and Meta-Analyses (PRISMA) reporting standards to ensure a comprehensive and reproducible synthesis of evidence. The search was performed using the following search terms, adapted for each database: (“radiomics” OR “dosiomics” OR “ultrasomics”) AND (“cardiotoxicity” OR “cardiac dysfunction” OR “CTRCD”) AND (“cancer” OR “oncology” OR “neoplasm”) AND (“detect” OR “predict”). Full details of the database-specific search strings are provided in the Supplemental Appendix. We also included articles cited in select publications.

**Figure 1:**
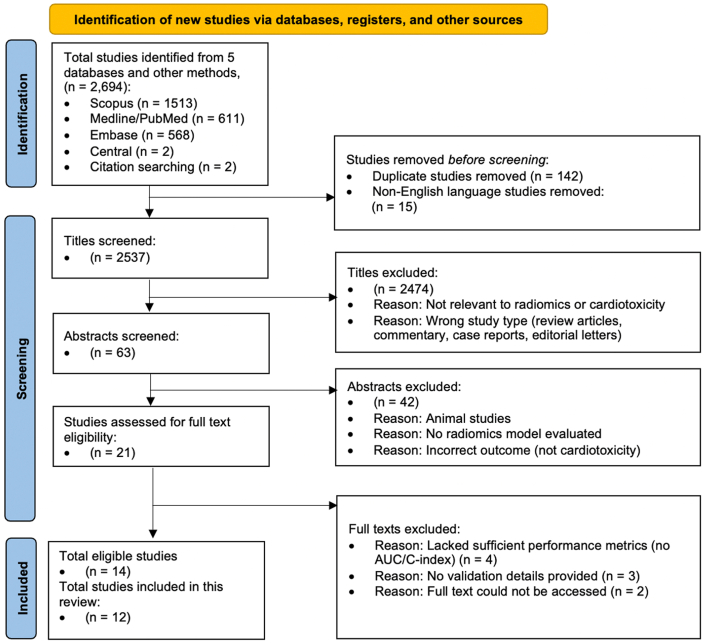
**Flow Diagram of Selection Strategy** AUC = area under the receiver operating characteristic curve.

Inclusion criteria were primary research studies and abstracts that explicitly reported on the development, performance, or evaluation of radiomics-based models for the detection or prediction of cardiotoxicity in patients receiving cancer treatment. All included articles were published in English. As this is not a systematic review, study inclusion was determined by consensus among authors. Exclusion criteria were studies that were on animals, not in English, or lacking sufficient data/relevance on radiomic model performance in patients with cancer. The final eligibility of each study was assessed based on the presence of clear performance metrics, details of model validation, and its direct relevance to the research question. Of the 14 studies initially identified as eligible, 2 were excluded on full-text review as they did not report radiomic model performance for cardiotoxicity endpoints, yielding 12 studies for inclusion (Table 2). We acknowledge that several included studies are available only as conference abstracts or study protocols, which limits the depth of methodological assessment; however, given the nascent state of this field, their inclusion provides a more comprehensive view of the current research landscape. The selection process is detailed in the PRISMA flow diagram (Figure 1).

**Table 2:** Summary of Current Literature Exploring Radiomics in the Detection and Prediction of Cardiotoxicity in Cancer Patients

Study Name	First Author (Year)	DOI	Study Design	Scanner and Imaging Settings	Cohort Population	Imaging Modality	Cardiotoxicity Endpoint	Best Performing Model	Detection/Prediction	RQS
Clinical Study of Post-Chemotherapy Cardiotoxicity in Breast Cancer Patients Based on Ultrasound Radiomics.^73^	Xia et al (2024)	https://doi.org/10.1136/openhrt-2023-002493	Retrospective	Philips EPIQ 7C	Breast cancer (n = 208)	Echocardiography	New relative decline in GLS ≥15%	Clinical + radiomics model (AUC = 0.87)	Detection	11
Association of echocardiographic radiomics-based features with cardiotoxicity effect in breast cancer patients from the CARDIOCARE project.^75^ (Abstract)	Manikis et al (2025)	https://doi.org/10.1093/ehjci/jeae333.028	Prospective	Deep neural network auto-segmentation	Breast cancer (n = 28)	Echocardiography	Changes in LVEF, GLS 3 mo post-treatment	-	Detection	4
Understanding cardiac events in breast cancer (UCARE): pilot cardio-oncology assessment and surveillance pathway for breast cancer patients.^81^ (Protocol)	Sacco et al (2023)	https://doi.org/10.1101/2023.12.15.23300055	Prospective	N/A (study protocol)	Breast cancer (n = 100 Planned)	Echocardiography	Acute therapy-related cardiotoxicity	-	Prediction and Detection	-
Prediction of heart failure and all-cause mortality using cardiac ultrasomics in patients with breast cancer.^77^	Hathaway et al (2024)	https://doi.org/10.1007/s10554-024-03101-2.	Retrospective	Philips and General Electric scanners	Breast cancer (n = 134)	Echocardiography	Incident Heart Failure	Ultrasomics + demographics (C-index = 0.77)	Prediction	7
Machine learning based radiomics model to predict radiotherapy induced cardiotoxicity in breast cancer.^80^	Talebi et al (2024)	https://doi.org/10.1002/acm2.14614	Prospective	Philips EPIQ-7	Breast cancer (n = 83)	Echocardiography	10% LVEF reduction to <55%	Dosimetrics + clinical + radiomics (AUC = 0.97)	Prediction	12
Acute coronary event (ACE) prediction following breast radiotherapy by features extracted from 3D CT, dose, and cardiac structures.^95^	Choi et al (2023)	https://doi.org/10.1002/mp.16345	Retrospective	3D FC DenseNet auto-segmentation	Breast cancer (n = 84)	CT	Acute coronary events	Deep learning model (96% accuracy)	Prediction	14
Prediction of Radiation Therapy Induced Cardiovascular Toxicity from Pre-treatment CT Images in Patients with Thoracic Malignancy via an Optimal Biomarker Approach.^76^ (Abstract)	Long et al (2025) (Abstract)	https://doi.org/10.1016/j.acra.2025.01.012.	Retrospective	Philips Gemini TF Big Bore Scanner (120 kVp)	Breast, lung, lymphoma (n = 125)	CT	Decrease in LVEF ≥10% or GLS ≥15%	Radiomics (88% accuracy)	Prediction	7
Radiomics Analysis on Computed Tomography Images for Prediction of Chemoradiation-induced Heart Failure in Breast Cancer by Machine Learning Models.^94^	Ansari et al (2024)	https://doi.org/10.4103/jmss.jmss_51_24	Retrospective	SwinUNETER deep learning segmentation	Breast cancer (n = 54)	CT	Heart failure 3 years post-therapy	Radiomics + patient age (AUC = 0.98)	Prediction	9
Combining dosiomics and machine learning methods for predicting severe cardiac diseases in childhood cancer survivors: the French Childhood Cancer Survivor Study.^96^	Bentriou et al (2024)	https://doi.org/10.3389/fonc.2024.1241221	Retrospective	-	Varied cancers (n = 7,367)	CT/Dosiomics	≥Grade 3 Cardiac disease	Dosiomics (C-index = 0.792)	Prediction	10
Novel Functional Radiomics for Prediction of Cardiac Positron Emission Tomography Avidity in Lung Cancer Radiotherapy.^85^	Choi et al (2024)	https://doi.org/10.1200/CCI.23.00241	Retrospective	Standard 18F-FDG PET/CT	Lung cancer (n = 209)	FDG-PET/CT	Classification of metabolic uptake patterns	9-feature radiomic signature (95% accuracy)	Detection	16
Reduced Homogeneous Myocardial [18F]FDG Uptake in Routine PET/CT Studies as an Early Indicator of Chemotherapy-Induced Cardiotoxicity.^6^	Palomino-Fernández et al (2024)	https://doi.org/10.3390/app142411653	Retrospective	Siemens Biograph 6 and GE Discovery MI	Solid or hematological cancers (n = 29)	FDG-PET/CT	LVEF <50%	-	Detection	5
Prediction of Radiation-Induced Microvascular Damage After Left Breast Cancer Radiation Therapy by Using Machine Learning and Cardiac SPECT Radiomics Features.^86^	Askari et al (2022)	-	Retrospective	-	Breast cancer (n = 32)	SPECT MPI	RMID 6 months post-RT	Radiomics (AUC = 0.85)	Prediction	8

Summary of current literature exploring radiomics in the detection and prediction of cardiotoxicity in patients with cancer.

AUC = area under the curve; CMR = cardiovascular magnetic resonance; CT = computed tomography; CTRCD = cancer therapy-related cardiac dysfunction; MPI =myocardial perfusion imaging; PET = positron emission tomography; RMID = radiation-induced microvascular damage; RT = radiotherapy; other abbreviations as in Table 1.

### The radiomics workflow

In radiomics, the translation of images into predictive data follows a highly structured, multistage workflow consisting of 5 key stages: 1) image acquisition; 2) image segmentation; 3) image preprocessing; 4) feature extraction; and 5) feature selection and model building.^24^ Each step is critical to ensuring the robustness, reproducibility, and clinical utility of the final model. However, each step also presents unique challenges, particularly when applied to the dynamic environment of the heart.^23^^,^^24^

### Image acquisition and reconstruction

**Figure 2:**
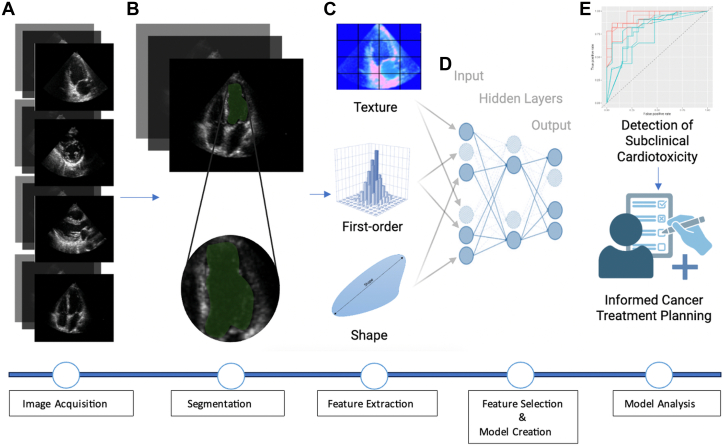
**Radiomics Workflow of Model Creation** This process begins with the acquisition and preprocessing of high-quality medical images, from which 3-dimensional regions of interest (ROIs) are segmented. The segmentation mask typically targets the left ventricular myocardium (LVM) rather than the cavity, as the myocardial tissue is the primary target of cardiotoxicity-related changes. Numerous quantitative features describing various properties of the ROI are extracted. Robust features are then used to build and validate a predictive/detective model, which can be integrated with clinical data for enhanced performance.^23^

The workflow begins with the acquisition of images from modalities, such as CT, MRI, PET, and ultrasound (Figure 2A).^28^ Acquisitions are typically stored in digital imaging and communications in medicine format and accessed via a clinical picture archiving and communication system or public repositories like the “Open-Source Imaging Consortium” and “The Cancer Imaging Archive”.^29^^,^^30^ A central challenge in the clinical translation of radiomics is interobserver variability, as identical myocardial tissue can yield divergent numerical texture values depending on the ultrasound system utilized (eg, Philips vs GE), a critical issue in multicenter studies.^29^^,^^30^ Furthermore, CT radiomic feature stability is directly impacted by the choice of reconstruction kernel (sharp vs smooth), which significantly impacts voxel texture, introducing a significant source of variability.^31^ Similarly, in PET imaging, variations in reconstruction algorithms and postreconstruction smoothing (Gaussian filter width) directly impact the final standardized uptake values (SUVs) and the textural appearance of the myocardium.^32^ In CMR, the qualitative late gadolinium enhancement protocol is different from the quantitative T1 mapping protocol.^30^ Late gadolinium enhancement and T1 mapping protocols produce fundamentally different radiomic fingerprints, and a model trained on one protocol cannot be validly applied to the other, thus altering the radiomic “fingerprint” and reducing reproducibility.^30^

In addition, cardiac images are uniquely susceptible to motion artefacts from the heartbeat. Without proper electrocardiogram (ECG)-gating, these movements can blur the fine details of the myocardial wall, compromising the reliability of the very texture features meant to detect subtle, diffuse changes like early cardiac fibrosis.^33^ To mitigate these issues, advanced computational techniques are now applied during the image reconstruction phase. For retrospective studies where protocol variation is present, postacquisition harmonization techniques like ComBat batch correction are often necessary to statistically adjust for scanner-specific effects in the data, although this cannot fully compensate for poor initial image quality.^29^^,^^30^^,^^34^ Beyond ComBat, other harmonization approaches include deep learning-based style transfer methods and latent feature extraction techniques, further reducing scanner-specific effects.^31^ In addition, appropriate image discretization before textural feature calculation has been demonstrated to substantially improve reproducibility across different acquisition settings.^35^ Future studies should adopt standardized reporting frameworks, particularly the recently proposed Radiomics Quality Score (RQS) 2.0 and the CheckList for EvaluAtion of Radiomics research checklist, which provide more comprehensive guidance for ensuring methodological transparency and reproducibility in radiomics research.^36^^,^^37^

### Image segmentation

Following image acquisition, a region of interest (ROI) is precisely delineated in a process known as segmentation.^28^ The choice of segmentation target is a critical variable: options include the whole heart, whole cardiac chamber including the blood pool, or isolated myocardium only. Although myocardial-only segmentation is more challenging, particularly in echocardiography, 3D imaging modalities such as CT and CMR enable precise delineation of the left ventricular myocardium, which is the most clinically relevant target for cardiotoxicity assessment.^28^ In 2D imaging, such as a single ultrasound frame, this delineated area is a single ROI. In 3D imaging modalities (PET/CT), stacking 2D ROIs across axial slices forms a 3D volume of interest (VOI).^38^ The traditional approach is manual segmentation, a labor-intensive process where a clinical expert contours around the ROI on each 2D slice (Figure 2B). This is performed using open-source platforms like 3D Slicer and ITK-SNAP, and ImageJ/Fiji, or commercial packages such as MIM software.^24^ Manual segmentation is time-consuming and prone to interobserver variability, compromising the reproducibility of radiomic features. Interobserver variability has the greatest impact on textural features, as a minor contour shift fundamentally redefines the set of neighboring voxels along the entire border, destabilizing the metric.^39^

This limitation has driven a shift toward semiautomated and fully automated methods leveraging AI.^40^ The current gold standard for cardiac segmentation involves deep learning models, particularly Convolutional Neural Networks like the (nn)U-Net model, which is a highly effective, self-adapting framework.^41^ A key limitation, however, is that these automated models lack generalizability, performing poorly when validated on external data sets, a critical challenge for widespread clinical implementation.^30^ Evaluating segmentation consistency is therefore crucial. As Akramova and Watanabe^42^ indicate, common geometric metrics like the Dice Similarity Coefficient can have low sensitivity, showing high agreement (Dice Similarity Coefficient >0.8) even when clinically relevant differences within the contour exist. They propose that the intraclass correlation coefficient (ICC) of the radiomic features themselves serves as a more reliable and sensitive measure of feature concordance, as small alterations in a segment contour can lead to large changes in texture features, impacting the final model’s reliability.^23^ Features with an ICC below a predefined threshold are considered nonreproducible and are excluded from the analysis of a study.^23^ The predefined threshold is set by a study’s authors before analysis, based on established statistics. Common ICC thresholds are often >0.75, with 0.75 to 0.90 signifying good reproducibility, and any features with an ICC >0.90 correspond to excellent reproducibility.^43^

### Image preprocessing

Once an ROI/VOI is segmented, preprocessing steps are applied to minimize technical variability. These include resampling voxels to a uniform isotropic spacing such as 1 × 1 × 1 mm^3^.^44^ Nonuniformity correction algorithms such as N4ITK and intensity normalization are then applied to ensure features reflect tissue biology rather than scanner-specific artefacts.^45^, ^46^, ^47^, ^48^

Image discretization, the grouping of continuous intensity values into a fixed number of bins, reduces noise but requires careful standardization, as bin width directly affects feature values.^29^^,^^35^^,^^49^ Notably, appropriate discretization has been shown to improve the reproducibility of textural features across different scanners and acquisition protocols.^49^ Finally, mathematical filters such as Wavelet or Laplacian of Gaussian transforms can be applied to enhance edges at different spatial scales, potentially capturing the subtle microarchitectural changes seen in early fibrosis.^50^

### Feature extraction

Feature extraction is a critical step of the radiomics workflow, where algorithms convert the segmented, preprocessed ROI/VOIs into a high-dimensional data set. This is performed using software like PyRadiomics, IBEX, or LIFEX.^51^ However, high variability within these software warrants the need for standardization, such as the “Image Biomarker Standardization Initiative”.^52^ Features fall into several categories including first-order (histogram) features, shape-based features, and texture (higher-order) features.^53^ First-order features describe signal intensities within the ROI/VOI.^53^ These features are calculated from the image histogram and include metrics like mean, skewness, kurtosis, and entropy. Within PET/CT imaging, SUVs are the first-order features commonly used to reflect cardiotoxicity.^6^ First-order features can track shifts in tissue density that may reflect myocardial edema.

Shape-based features, such as sphericity and surface-to-volume ratio, capture morphological irregularities indicative of pathology.^54^^,^^55^ These features quantify the morphological remodeling that characterizes heart failure and early cancer therapy-related cardiotoxicity. For example, this could provide an objective, reproducible assessment of myocardial fibrosis that characterizes progressive cancer therapy-related cardiotoxicity.^54^

Texture features quantify the spatial arrangement and relations between neighboring voxels, far more accurately than the detectable resolution of the human eye.^56^ Tissue patterns evaluated using matrices, like the gray level co-occurrence matrix (GLCM), assess the probability of 2 voxel intensities occurring at a specific distance and direction.^53^ From this, features like contrast and correlation are derived. Similarly, the gray level run length matrix (GLRLM) quantifies the distribution of consecutive voxels of identical intensities along particular directions.^56^ Additional features include the gray level size zone matrix and neighboring gray tone difference matrix.^57^

Alternatively, deep radiomics uses Convolutional Neural Networks to automatically learn and extract features directly from image data. Unlike “hand-crafted” radiomics, such as GLCM/GLRLM, which rely on predefined mathematical formulas and require precise segmentation, deep radiomics can capture complex, nonlinear patterns, potentially identifying signatures that no individual texture matrix would generate.^53^ However, deep radiomics requires substantially larger training data sets than hand-crafted radiomics, a significant challenge given the small cohort sizes characteristic of cardiotoxicity research. Furthermore, the learned features often lack the biological interpretability of textural features, making it difficult to link radiomic signatures to specific pathophysiological processes such as fibrosis or inflammation.^53^ These features collectively provide a quantitative measure of tissue heterogeneity that may correspond to early fibrosis, extracellular matrix remodeling, or inflammatory infiltration. Although advanced modalities like CMR with late gadolinium enhancement and native T1/T2 mapping are designed to detect these very processes, such subtle or diffuse alterations are difficult to visually quantify, thus may be missed or misinterpreted, hence can fall below the threshold for what is considered a positive finding on a standard read^53^^,^^56^ (Figure 2C).

### Feature selection

The extraction process can generate over 1,000 radiomic features, many of which are redundant, creating a high risk of model overfitting.^58^ Overfitting underscores the robustness of the model and its clinical utility.^30^ Thus, feature selection is a critical step to identify a robust subset of relevant features. The goal is to distil thousands of measurements into a stable and clinically meaningful “cardiotoxicity signature.” Feature selection occurs through 3 main methods: filter (ranking based on their intrinsic statistics), wrapper, and embedded methods.^23^ For cardiotoxicity research, this often involves the Mann-Whitney *U* test to identify radiomic features whose distributions differ significantly between patients who do and do not develop cancer therapy-related cardiotoxicity.^23^ Critically, such supervised filter methods must be performed exclusively within the training data set to prevent information leakage between model development and validation, a methodological requirement that is inconsistently reported in the current literature. Furthermore, this approach assesses features in isolation and may fail to identify complex interactions between markers that, together, could signal early cardiotoxicity.

Wrapper methods, such as Recursive Feature Elimination, use model performance to iteratively select optimal feature subsets.^23^ Although powerful, they are computationally intensive and risk overfitting in small cohorts.^58^ Embedded methods such as least absolute shrinkage and selection operator regression integrate feature selection directly into the model training process, producing a sparse and clinically interpretable signature.^23^^,^^59^ Other examples of embedded methods include Gradient Boosted Decision Trees, which favors features that are better at correcting errors, effectively ranking, and selecting features as part of its learning process^23^ (Figure 2D).

### Model building and validation

Once an optimized feature set is selected, a model is built to detect or predict a known cardiotoxicity endpoint, such as a drop in LVEF or the onset of heart failure. These models are either diagnostic (detecting existing cardiotoxicity) or predictive (estimating future cardiotoxicity risk). This typically involves supervised learning, where a model is trained to learn the rules that optimally predict a known clinical outcome from the input of features.^60^ This is performed using statistical software, such as Python, with scikit-learn or R, which implement a range of machine learning classifiers.^61^ Common model classifiers include Support Vector Machines (SVMs), Random Forests (RFs), and Extreme Gradient Boosting.^23^ Each machine learning model offers unique advantages: SVMs are highly effective at separating complex imaging data into distinct categories, RFs combine multiple predictions to improve overall reliability, and Extreme Gradient Boosting continuously refines its accuracy by learning from its previous errors.^23^^,^^62^

Rigorous model validation is essential for clinical translation. Internal validation via k-fold cross-validation ensures model stability, whereas discriminative performance is evaluated using the area under the receiver operating characteristic curve (AUC), with values above 0.8 generally considered clinically useful.^23^^,^^30^^,^^63^ External validation on an independent cohort is the definitive test of generalizability. The entire process must be transparently documented according to established reporting guidelines^61^ (Figure 2E). The RQS is a 36-point system assessing 16 key dimensions of methodological rigor, weighing feature reproducibility, open-science data sharing, and external validation as critical benchmarks. More recently, the RQS 2.0 and the CheckList for EvaluAtion of Radiomics research have been proposed to further strengthen reporting standards, addressing limitations of the original RQS such as the lack of emphasis on clinical utility and calibration.^36^^,^^37^

### From technical workflow to a clinical biomarker

The systematic workflow of radiomics culminates in the generation of quantitative “radiomic signatures”, which are comprehensive, data-driven fingerprints of tissue characteristics that provide precise and objective clinical insights.^64^ The use of these signatures has been validated across multiple cancer types in oncology for improving tumor diagnosis and predicting treatment response, as seen in the CT-based radiomics study of non–small cell lung cancer, with a robust AUC of 0.75, and another signature associated with response to immune checkpoint inhibitors, achieving an AUC of 0.67 in external validation.^65^^,^^66^ Its utility is now expanding into cardiology, where a coronary CT angiography radiomic signature independently predicted major adverse cardiac events (*P* = 0.005) and improved risk stratification for myocardial infarction (C-statistic 0.74) in the SCOT-HEART trial.^67^^,^^68^ Within the field of radiomics are specialized subfields of dosiomics and ultrasomics, which extract features from 3D dose distributions of radiotherapy and ultrasonography, respectively.^69^^,^^70^ The potential of this approach lies in its ability to identify elusive pathophysiological changes in tissue that are beyond the limits of conventional visual perception. These signatures could improve the detection and prediction of cardiotoxicity in patients with cancer, across multiple imaging modalities, thereby potentially facilitating earlier recognition (Central Illustration).^71^

**Central Illustration:**
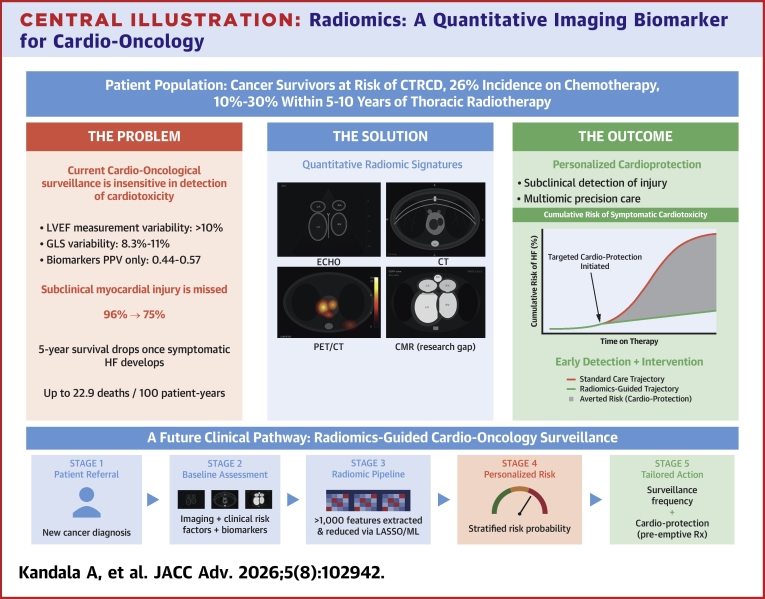
**Radiomics: A Quantitative Imaging Biomarker for Cardio-Oncology** The radiomics pipeline transforms routine cardiac imaging into high-dimensional quantitative signatures. Across 12 published studies in echocardiography, CT and PET/CT, radiomic models have achieved internal AUCs of 0.77 to 0.97 for the detection or prediction of cancer therapy-related cardiotoxicity. Clinical translation now requires prospective multicenter validation, broader cancer populations beyond breast, dedicated CMR investigation, and adherence to updated reporting standards (RQS 2.0, CLEAR). CMR = cardiac magnetic resonance imaging; CT = computed tomography; CTRCD = cancer therapy-related cardiac dysfunction; ECHO = echocardiography; GLS = global longitudinal strain; HF = heart failure; LASSO = least absolute shrinkage and selection operator; LVEF = left ventricular ejection fraction; ML = machine learning; PET = positron emission tomography; PPV = positive predictive value.

### Radiomics in cardiac imaging

#### Radiomics in echocardiography

**Figure 3:**
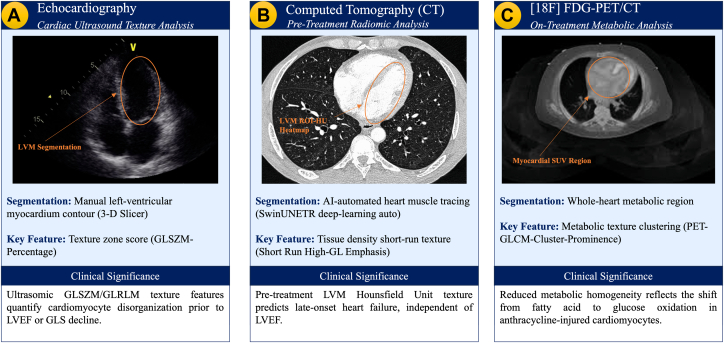
**Clinical Significance of Radiomic Analysis in Cardiac Imaging** (A) Ultrasomic analysis achieved from myocardial segmentation, texture feature extraction, and model output. This can aid detection of cardiomyocyte disorganization before cardiac decline. (B) CT scans often undergo deep-learning auto segmentation to extract radiomic features, which, when combined with HU texture, can predict late-onset heart failure. (C) PET/CT radiomics are extracted from myocardial SUV data and whole-heart metabolics, with cardiac homogeneity showing clinical significance (PET/CT, 95% accuracy).^73^^,^^85^ CT = computed tomography; FDG = Fluorodeoxyglucose/18F-FDG; LVEF = left ventricular ejection fraction; GL = gray Level; GLCM = gray level co-occurrence matrix; GLRLM = gray level run length matrix; GLS = global longitudinal strain; GLSZM = gray level size zone matrix; HU = Hounsfield units; LVM = left ventricular myocardium; PET = positron emission tomography; ROI = region of interest; SUV = standardized uptake value.

The literature exploring the use of ultrasomics, radiomics applied to echocardiography, demonstrates a promising but emerging field characterized by significant methodological heterogeneity (Figure 3A). Current evidence suggests ultrasomics can quantify subvisual myocardial texture to detect/predict cardiotoxicity, although models often lack validation. Their clinical translation is hampered by the inherent technical challenges of ultrasound imaging, such as motion artefact, where both cardiac and respiratory motion can degrade image quality.^72^ Furthermore, the evidence base is narrow, with the literature almost exclusively focused on cohorts of patients with breast cancer (Table 2). This significantly limits the external generalizability of these findings to other malignancies, different therapeutic regimens, and patterns of cardiac exposure. Indeed, of the 12 studies included in this review, 9 were conducted exclusively in breast cancer cohorts, and all echocardiography-based radiomics studies focused solely on breast cancer populations. This concentration of evidence introduces a substantial selection bias, as the pathophysiology of cardiotoxicity may differ across cancer types and treatment regimens. For example, the cardiotoxic mechanisms of anthracyclines in breast cancer differ from those of immune checkpoint inhibitors in melanoma or targeted therapies in hematological malignancies, potentially yielding distinct radiomic signatures.^9^ Future research must prioritize broader, more diverse patient populations to ensure that radiomics-derived biomarkers are robust and transferable across the oncologic spectrum.

### Detecting cardiotoxicity

Ultrasomics has demonstrated proficiency in detecting subclinical cardiotoxicity by quantifying the heterogeneity of the myocardial tissue.^73^ The ultrasomic texture features with the greatest discriminative utility, particularly gray level size zone matrix (GLSZM) and GLRLM metrics, reflect the progressive spatial disorganization of myocardial echogenicity as cardiomyocyte apoptosis and patchy fibrotic replacement accumulate under anthracycline injury.^26^^,^^73^

Xia et al^73^ reported that a clinical and ultrasomic data-based model was superior for diagnosing asymptomatic cardiotoxicity.^73^ Echocardiograms were acquired on a Philips EPIQ 7C scanner, and the myocardium was manually segmented in the parasternal long-axis view at end-diastole using 3D-Slicer software. The Philips EPIQ 7C scanner ensures a high degree of technical consistency. However, the resulting model will therefore be highly optimized for Philips-specific image characteristics, such as proprietary speckle-reduction and beamforming algorithms, and its performance on images from other vendors is unknown, a key barrier to generalizability.^74^ From an initial set of 852 features, a combination of minimum redundancy maximum relevance and least absolute shrinkage and selection operator regression was used to select 14 key textural features from the GLSZM, GLRLM, and GLCM families.^73^ The “GLSZM-Percentage” feature showed the greatest utility in quantifying the homogeneity of myocardial speckle patterns, providing a direct measure of tissue disruption, with the greatest ICC of 1.431 in the final binary logistic regression model.^73^ However, the study received a modest RQS of 11 of 36, reflecting its retrospective design, lack of external validation, and use of manual segmentation, thereby introducing interobserver variability and limiting reproducibility.^73^

Corroborating the approach, Manikis et al^75^ conducted a prospective study, the CARDIOCARE project, presented as an abstract, on a small cohort of 28 patients, identifying 40 significant textural radiomic features on 3-month follow-up echocardiographic scans that could discriminate cardiotoxic outcomes.^75^ These outcomes included changes in LVEF and GLS. In contrast to the manual approach of the previous study, this work used a pretrained deep neural network for automated segmentation of the apical 4-chamber and 2-chamber views. The primary advantage of this approach is the elimination of interobserver variability. Their analysis revealed that the most informative features for detecting established cardiotoxicity at 3 months were predominantly derived from the apical 4-chamber view at end diastole.^75^ However, 1 limitation of this study was the short follow-up period of only 3 months, limiting the assessment of delayed cardiotoxicity.^75^^,^^76^ Although this study was limited by a small sample size, it emphasizes that changes in myocardial heterogeneity may serve as a detectable marker of early cardiotoxicity. This study received an RQS of 4 of 36, a direct result of its exploratory nature with no validation cohort, model calibration, or comparison to the current clinical gold standard, of echocardiographic surveillance.

### Predicting cardiotoxicity

The predictive capabilities of ultrasomics were explored by Hathaway et al^77^ in a cohort of 134 patients with breast cancer. The study demonstrated that combining pretreatment textural ultrasomic features with demographic data significantly improved discriminative capacity, increasing the model’s C-index for mortality from 0.65 to 0.78 and for heart failure from 0.60 to 0.77. Images were acquired in the Philips and General Electric scanners, and features were extracted from the manually traced parasternal long-axis view using the LIFEx software.^77^ However, a major limitation of the study’s acquisition protocol is the lack of harmonization techniques to account for intervendor variability in image processing and texture. This introduces potential domain shift, as the model may be learning scanner-specific artifacts rather than true radiomic signatures of cardiotoxicity. Crucially, their model was not a simple addition of features as they used a Cox Proportional Hazards model in R software to combine the top 10 most predictive texture features into a single composite risk score.^77^ However, this model was developed in a single-center cohort with no external validation and was trained on a demographically homogenous population that was 95% Caucasian.^77^ This limits generalizability across different ethnicities, who may possess different baseline cardiovascular risk profiles or biological responses to therapy.^78^ A narrow study demographic may also risk spurious correlation, as the radiomics model may identify features that act as proxies for ethnicity, such as baseline differences in myocardial tissue texture between ethnicities, rather than the true signatures of cardiotoxicity.^79^ These methodological shortcomings resulted in an RQS of 7 of 36.

The prospective cohort study design Talebi et al^80^ represents a significant improvement over previous retrospective analyses by reducing the risk of selection bias. They studied 83 patients with breast cancer undergoing radiotherapy, generating a multimodal model with an AUC of 0.97 for predicting radiotherapy-induced cardiotoxicity, highlighting the utility of integrating radiomics with clinical data, although this finding is derived from a relatively small cohort.^80^ Images were acquired on a Philips EPIQ-7 machine, and features were extracted using the LIFEx software from the short-axis views.^80^ Their model-building process involved using recursive feature elimination for selection and an SVM for classification. Analysis revealed that the most significant radiomic predictors were seen in the end-systole phase of the cardiac cycle.^80^ These predictors were again textural features from the GLRLM and GLSZM families. This study further highlights the potential importance of analyzing the myocardium under maximum contraction for prognostic purposes.^80^ Despite its prospective design, the study achieved an RQS of 12 of 36, due to its small sample size (n = 83), lack of external validation, and use of manual segmentation.^80^ However, this study’s strict exclusion criteria, which removed patients with a prior history of heart disease, severe hypertension, and other comorbidities, created a highly selected cohort that may not reflect the more complex patient populations encountered in routine clinical practice. The presence of a detailed protocol of future radiomic trials, as seen in the “UCARE” (Understanding cardiac events in breast cancer) pilot study, conveys the field’s maturation and its recognition of the need for other large-scale, prospective, multicenter studies to definitively validate these models.^81^

Overcoming the limitations identified across echocardiography-based radiomic studies requires coordinated methodological reform at several levels. This can be mitigated through: 1) prospective multivendor acquisition with matched imaging parameters; 2) postacquisition harmonization using ComBat batch correction or deep learning-based style transfer; and 3) image biomarker standardisation initiative-compliant feature extraction to ensure textural metrics are computed consistently across platforms.^29^^,^^30^^,^^36^ Manual myocardial segmentation, which underpins 4 of the 5 included echo studies, introduces interobserver variability that fundamentally destabilizes texture metrics. Thus, future studies should mandate automated convolutional neural network segmentation with ICC-based reproducibility reporting to exclude unstable features before model training.^23^^,^^41^

### Radiomics in PET/CT and CT

PET and CT, central to routine oncologic staging and radiotherapy planning, represent data-rich frontiers for cardiotoxicity radiomics (Figures 3B and 3C).

### Detecting cardiotoxicity: the metabolic signature on PET/CT

On-treatment PET/CT scans detect a unique metabolic signature of cardiotoxicity characterized by a myocardial shift toward metabolic homogeneity. A retrospective study of 29 cancer patients by Palomino-Fernández et al^6^ with solid or hematological malignancies, found that those who developed cancer therapy-related cardiotoxicity showed significantly lower myocardial SUVs (4.30 vs 7.10; *P* = 0.025) and increased metabolic homogeneity on midtreatment scans. The reduced metabolic homogeneity and lower SUV observed in cardiotoxic patients is biologically plausible as healthy cardiomyocytes preferentially oxidize fatty acids, but anthracycline-induced mitochondrial injury shifts substrate use toward glycolysis in viable cells, whereas progressive cardiomyocyte loss and fibrous/adipose replacement generate spatially heterogeneous Fluorodeoxyglucose/18F-FDG activity.^82^

The clinical relevance of this finding is limited by the study’s small size, retrospective design, and lack of control for factors such as diet, which is known to influence myocardial uptake of glucose in PET/CT scans.^6^ Furthermore, this study primarily examined SUV-based metrics rather than high-throughput textural feature extraction, and as such may not fully represent the high-dimensional nature of a radiomics approach. This study achieved an RQS of 5 of 36. In addition, initial PET images were acquired on different scanner hardware (Siemens Biograph 6 and GE Discovery MI).^6^ The difference in crystal composition between the 2 scanners results in different timing resolution of images.^83^ This is critical for an advanced reconstruction technique called “time-of-flight”.^84^ Improved timing resolution results in an image with a greater signal-to-noise ratio and sharper contrast, particularly affecting the extraction of texture features, such as GLCM, thus reducing the reproducibility of the study.^83^

Choi et al^85^ established a 9-feature signature for classifying these metabolic states in 202 lung cancer patients with 95% accuracy. This study established a robust, automated method for classifying these cardiac metabolic states using an Extra Trees Classifier developed with the Tree-based Pipeline Optimization Tool AutoML framework in Python. This was accomplished using an optimized 9-feature radiomic signature extracted with PyRadiomics, which included first-order statistics like “Pet-2D-Skewness” and textural features like “PET-GLCM-Cluster-Prominence”.^85^ A key strength of this study was its use of 2 independent external cohorts for validation. However, this further revealed a core challenge of radiomics, as the model’s accuracy dropped from 91.9% on 1 external data set to 80.3% on an older cohort,^85^ a stark illustration of how sensitive these models are to real-world variations in image quality and acquisition. Askari et al employed regularization-based machine-learning algorithms that use rest/stress myocardial perfusion imaging in 32 patients with breast cancer. A total of 115 radiomic features were extracted to estimate radiation-induced microvascular damage after 6 months of radiotherapy. The elastic spontaneous coronary artery dissection-penalized SVM model achieved the greatest AUC of 0.85.^86^

A fundamental methodological limitation of applying radiomics to routine oncological FDG-PET/CT is the lack of myocardial metabolic suppression. Unlike routine oncological scans, dedicated cardiac protocols use high-fat, low-carbohydrate dietary preparation, with 72-hour ketogenic diets achieving 97% complete myocardial suppression, vs only 52% with 18-hour fasting.^87^ Even with optimal protocols, physiological myocardial FDG uptake persists in up to 20% of patients.^88^ Thus, myocardial FDG uptake in routine oncologic imaging is highly variable, making radiomic features derived only from the myocardium unreliable, as they may reflect transient metabolic states rather than pathology.^6^

To overcome this, radiomic analysis is increasingly shifting toward noncardiac thoracic structures, which possess more stable metabolic profiles. The aorta and carotid arteries represent critical targets, as radiomic quantification of these regions can aid the detection of vascular inflammation and injury, relevant to radiation-induced vasculopathy and accelerated atherosclerosis.^89^ FDG uptake in arterial walls correlates closely with macrophage infiltration within atherosclerotic plaques.^90^ Following thoracic radiotherapy, significant dose-dependent increases in aortic inflammation occur. In head and neck cancer patients, carotid artery FDG uptake increases 19% to 21% at 3 months postchemoradiotherapy, with human papillomavirus-positive tumors showing greater vascular inflammation.^91^ Focal increased myocardial FDG uptake occurs in 20% to 47% of patients following thoracic radiotherapy, particularly when cardiac volumes receive ≥20 Gy to ≥5 cm^3^, with persistent elevation up to 12 months.^92^

Furthermore, epicardial adipose tissue (EAT) offers a novel avenue for analysis. Changes in EAT metabolic activity and radiomic texture can serve as an indicator for pericoronary inflammation and adverse cardiometabolic remodeling, potentially offering a more reliable biomarker than the myocardium itself in routine oncologic imaging.^93^ EAT accumulation and inflammation are associated with coronary artery disease severity independent of visceral adiposity. Pericoronary adipose tissue attenuation on CT reflects adipocyte size and leukocyte infiltration, serving as an inflammation marker with prognostic value.^93^ EAT volume and metabolic activity correlate with plaque vulnerability in acute coronary syndrome, and quantitative EAT measures demonstrate incremental utility in predicting major adverse cardiovascular events when incorporated into AI models. In cardio-oncology, radiomics analysis of cardiac FDG uptake patterns has demonstrated 80% to 93% predictive accuracy for identifying pre-existing cardiac conditions and early cardiotoxicity risk biomarkers.^93^

To overcome PET/CT-based radiomic limitations, the variable myocardial FDG uptake that confounds routine oncological scans can be substantially reduced by implementing standardized low-carbohydrate, high-fat dietary protocols for 24 to 72 hours before scanning. As dedicated cardiac PET protocols employing ketogenic preparation achieve near-complete myocardial suppression (97% vs 52% with standard fasting), enabling more reliable myocardial texture extraction.^87^ In addition, adopting full high-throughput radiomic pipelines using PyRadiomics, rather than relying solely on first-order SUV metrics, will be essential to identify the texture-level information that could distinguish cancer therapy–related cardiac dysfunction from other metabolic cardiomyopathies.

### Prediction of cardiotoxicity: the power of CT

Pretreatment CT scans are a promising source for predictive models. In a retrospective study of 125 patients with thoracic malignancy, Long et al used pre-treatment noncontrast CTs, acquired on a Philips Gemini TF Big Bore Scanner (120 kVp).^76^ This abstract showcased their “Optimal Biomarker” approach, identifying a combination of 5 GLCM-based texture features from the left ventricle that predicted cardiotoxicity with an accuracy of 0.88.^76^ A significant limitation of their workflow was the reliance on manual segmentation using the CAVASS software, reducing the reproducibility of the final model.^76^ This, alongside the study’s single-center design and lack of external validation, resulted in a low RQS of 7 of 36. Notably, Long et al^76^ used noncontrast CT images acquired at 120 kVp, highlighting the importance of specifying imaging parameters, as factors including contrast administration, ECG-gating, and kilovoltage settings substantially affect image quality, applicable segmentation methods, and the feasibility of radiomics.

Similarly, a model by Ansari et al predicted late-stage heart failure with an AUC of 0.98.^94^ A technical strength of their workflow was the use of a SwinUNETER deep learning model for automated heart segmentation before building their RF model in Python.^94^ In their model, the radiomic feature “Short-Run-High-Gray-Level-Emphasis” was identified as a key predictor, alongside age and V5.^94^ However, this significant AUC was derived from a small cohort of only 54 patients with breast cancer undergoing chemoradiation, creating a high risk of model overfitting.^94^

Although noncontrast CT offers lower soft-tissue contrast compared to CMR, contrast-enhanced CT provides excellent myocardial delineation, and modern deep learning-based auto-segmentation methods, as utilized by Ansari et al,^94^ can further refine contour accuracy even in noncontrast acquisitions. Moreover, CT provides absolute attenuation values in Hounsfield Units (HUs) that are relatively standardized across scanners and manufacturers, a significant advantage over modalities such as MRI where signal intensity is inherently relative. These HU values additionally enable material characterization, such as the identification of calcification, fatty infiltration, and pericardial thickening, which are structural sequelae of radiation-induced cardiotoxicity that alter tissue density at the voxel level. This may produce CT radiomic signatures distinct from those seen in ischemic or hypertensive disease.^26^^,^^57^

To overcome CT-based radiomic limitations, protocol heterogeneity can be addressed by prespecifying acquisition parameters including reconstruction kernel, KvP, slice thickness, and contrast administration status before data collection begins. Where retrospective protocol variation is unavoidable, postacquisition harmonization using ComBat batch correction can statistically adjust for scanner-specific effects, although this cannot fully compensate for poor initial image quality.^34^^,^^76^ ECG-gated acquisitions should be prioritized where feasible, as ungated scans introduce cardiac motion artefact that directly destabilizes myocardial texture features.^33^^,^^76^ The limitation of radiation dose from repeated CT acquisitions is best overcome by integrating radiomic analysis into staging or surveillance CTs already scheduled as part of oncological care.

The next frontier of radiomics lies in applying deep learning and validating models in large-scale cohorts. Choi et al^95^ demonstrated the power of deep learning, developing a model using CT and dose maps that predicted acute coronary events with high test accuracy (96% cross-validation and 83% independent test set) and visually identified the left ventricle as a critical risk region, in breast cancer patients, post radiation-therapy. However, a large-scale study by Bentriou et al^96^ on over 7,000 childhood cancer survivors provided a crucial counterpoint, finding that for predicting late-onset disease (>20 years), complex dosimetric texture features offered no significant advantage over the simple “mean heart dose” (C-index ≈ 0.79). This highlights the importance of matching the feature to the biological endpoint. This finding strongly suggests that the underlying biological drivers, and therefore the detectable radiomic signatures for early-vs late-onset cardiotoxicity, may be fundamentally different.^96^ Furthermore, this study relied on phantom-based dose reconstructions to estimate historical cardiac exposures, a method prone to significant dosimetric error that may not accurately represent the true dose distribution received by the myocardium. The potential of novel imaging biomarkers in the field of cardio-oncology is summarized in the Central Illustration.

### The absence of CMR-based radiomics in cardiotoxicity

Despite CMR being the reference standard for myocardial tissue characterization and volumetric assessment, no published study to date has applied a radiomics workflow to CMR for the detection or prediction of cancer therapy-related cardiotoxicity. This is a notable gap, as CMR sequences such as native T1/T2 mapping and late gadolinium enhancement are specifically designed to quantify diffuse myocardial fibrosis, edema, and inflammation, the very processes that underlie cardiotoxic injury. The absence of CMR-based radiomics likely reflects several practical barriers: CMR’s higher cost and lower availability compared to echocardiography and CT, longer acquisition times that limit serial surveillance, and smaller existing data sets for model training. Nevertheless, the superior soft tissue contrast and quantitative nature of CMR parametric maps make it a compelling modality for future radiomic investigation. We advocate for greater deployment of CMR in cardio-oncology radiomics research, particularly given the potential for CMR-derived radiomic signatures to provide stronger biological grounding than those derived from other modalities.

### Future perspectives

Current literature establishes a proof-of-concept for cardiotoxicity radiomics but highlights significant limitations. Our structured narrative review demonstrated the following key findings: 1) existing studies in the field are limited both in number and methodology, with a mean RQS of 10.2 reflecting the largely retrospective nature of the studies, which require external validation; 2) despite these limitations, early results consistently show that quantitative imaging can identify myocardial heterogeneity that predicts or detects cardiotoxicity, often achieving AUC values of 0.80 to 0.97 in internal validation; and 3) integrating radiomic features with clinical or dosimetric variables improves the model performance in most studies, highlighting that multimodal or multiomic approaches hold potential for cardiotoxicity detection and prediction. Transitioning to clinical practice requires robust effectiveness-implementation trials, multiomics integration, and automated clinical decision support systems.

### Establishing clinical validity through effectiveness-implementation trials

With cardiotoxicity affecting up to 37.5% of patients undergoing moderate- or high-risk cancer therapy, the need for robust predictive tools is a pressing unmet need.^7^ Future development must move beyond the small, single-center retrospective analyses characterizing most studies in this review. Clinical adoption requires large-scale, prospective, multicenter hybrid effectiveness-implementation trials, evaluating both the effectiveness and real-world workflow integration.

The UCARE study protocol serves as a blueprint for this evolution, testing an entire surveillance pathway incorporating imaging data, serial biomarkers, and wearable device data.^81^ Such prospective, multicenter studies are essential for validating radiomic signatures across diverse populations, treatment regimens, and imaging hardware.^77^^,^^81^

These trials should also aim to refine modality-specific applications and establish optimal follow-up periods that are both evidence-based and clinically practical. Current evidence suggests [18F] FDG-PET/CT is a promising modality for real-time detection of on-treatment cardiotoxicity.^6^ In addition, pretreatment CT appears highly effective for late-stage heart failure prediction at a 3-year follow-up.^86^ However, these shorter timeframes are insufficient for capturing late-onset cardiotoxicity, seen in radiotherapy survivors. This is a critical gap highlighted by the time-dependent analysis of Bentriou et al^96^ performed in a cohort with a median follow-up of 30.2 years.

### Multimodal and multiomics integration

Radiomics provides a powerful macroscopic and microscopic view of tissue phenotype, but a key limitation of current models is their ability to explain why only a subset of patients develops cardiotoxicity. The next leap in predictive accuracy will come from integrating imaging radiomic data with other biological data streams.^97^ Prediction models combining clinical and genetic risk factors have already shown superior performance (AUC: 0.79) compared to models using clinical data only (0.69) or genetic risk factors only (0.67).^98^

The logical next step is to build comprehensive models that integrate radiomic features with genomic, proteomic, and metabolic features, accounting for patient demographics and clinical features.^99^^,^^100^ This multiomics approach, anticipated by the UCARE protocol’s plan to biobank patient samples, will create a more accurate and biologically informative risk model, moving the field beyond simple prediction toward a mechanistic understanding of individual susceptibility.^81^ However, this multiomic approach can also introduce substantial methodological and statistical challenges. Each “omic” modality has its own complex source of technical variability from different sequencing platforms in genomics to batch effects in proteomics, all of which need standardization. Furthermore, combining multiple high-dimensional data sets from small patient cohorts significantly increases the risk of discovering spurious correlations rather than true biological signals of cardiotoxicity.

Each imaging modality offers distinct advantages for cardiotoxicity radiomics. Echocardiography is widely accessible, cost-effective, and allows serial monitoring but is limited by operator dependence and intervendor variability. CT provides standardized HU values and excellent spatial resolution but involves ionizing radiation. PET/CT captures metabolic information but is confounded by variable myocardial FDG uptake. In addition, CMR offers superior soft-tissue characterization but is costly and less widely available. Understanding these modality-specific strengths and limitations is essential for guiding clinical decision-making about which imaging platform to deploy for radiomic surveillance. Most existing cardio-oncology radiomics studies have focused on a single imaging modality but have undertaken integration across various data domains (dosimetric and ultrasomic), underscoring a crucial next step for advancing the accuracy of models. To date, we have not found a published study combining radiomic features from more than 1 imaging modality within the same model for cardiotoxicity detection or prediction. Still, multimodal radiomics has demonstrated clear value in oncology and cardiovascular disease, where integration of various modalities helps capture information unavailable to any single technique.^101^^,^^93^ Extending this approach to cardio-oncology could improve outcomes and understanding of therapy-related cardiotoxicity.

### Clinical translation

For radiomics to make a true clinical impact, its insights must be delivered to clinicians in an automated, timely, and interpretable manner. Future model development must focus on the integration of models into real-time decision support systems, embedded within clinical workflow. An accurate classification of cardiotoxicity risk must be followed by clinical action, such as intensifying cardiac surveillance, with serial echocardiograms, or initiating evidence-based cardioprotective interventions.^102^ The successful implementation of radiomics will almost certainly require a cardio-oncology multidisciplinary team approach, involving oncologists, cardiologists, radiologists, and medical physicists to translate radiomic readings into actionable clinical recommendations.^103^

Ensuring global access to radiomics presents a fundamental challenge beyond creating a simple risk score for resource-deprived areas in lower-middle-income countries. The key challenge is that models are highly sensitive to the data on which they are trained. Thus, a model developed and validated on a Western population, as seen in the Hathaway et al study, may struggle to perform accurately when applied to minority populations due to underlying differences in genetics, comorbidities, and imaging hardware. Therefore, the development of globally accessible radiomics tools is contingent on dedicated, local data collection and model validation in diverse populations.

This translation also faces regulatory challenges. Any radiomic model intended for clinical decision-making will be classified as Software as Medical Device and must navigate regulatory pathways, meeting criteria for safety, effectiveness, and postmarket surveillance.^104^ This is particularly challenging for continuous learning algorithms, which evolve as they process new data, necessitating a framework of ongoing oversight between developers, regulators, and clinical end-users.^105^

Furthermore, reporting high discrimination (AUC) is insufficient for clinical adoption. Analysis of the RQS scores across the studies in this review reveals consistently poor performance in several key domains: external validation on independent datasets, prospective study design, open sharing of data and code, assessment of feature reproducibility, and comparison to the clinical gold standard. Systematic improvement across these specific domains is essential if the field is to produce robust, clinically translatable models. Further studies must also evaluate model calibration and decision curve analysis to demonstrate that radiomic models provide a net clinical benefit over current guideline-based risk scores, specifically in guiding clinical decisions.

## Conclusions

Radiomics offers opportunities to improve cardiotoxicity detection and prediction. Current evidence suggests that the quantitative measurement of myocardial heterogeneity, whether textural or metabolic, is a core radiomic signature. Characterizing the signatures of cardiac modeling could identify patients at risk of cardiotoxicity early to prevent the progression of cardiotoxicity in oncology patients. However, future prospective clinical trials and the integration with both multiomic and multimodal data could provide the robust validation needed to provide a crucial bridge from preclinical discovery to meaningful clinical translation at the patient’s bedside.

## Funding support and author disclosures

The authors have reported that they have no relationships relevant to the contents of this paper to disclose.
