# Supplementary material for: Radiomics for the Detection and Prediction of Cancer Therapy-Related Cardiotoxicity
Source: JACC Adv. 2026 Jul 1;5(8):102942. doi: 10.1016/j.jacadv.2026.102942 (PMC13355195; doi:10.1016/j.jacadv.2026.102942)
Supplement: Supplementary material [file mmc1.pdf]

Radiomics for the Detection and Prediction of Cancer Therapy-related Cardiotoxicity: A State-Of-The-Art Review

Abhinav Kandala BSc[a]*, Amar Rai, MBBS BSc[b,c]*, Rahul Penumaka MBBS MS[d], Nicholas S. Wilcox MD MHS[e,f], Benedicte Lefebvre MD[e,f], Paco E. Bravo MD[g], Michael G. Fradley MD[e]

Supplementary Appendix

Table of Contents

Medline (PubMed) Search Strategy 2

Scopus Search Strategy 3

Embase (Ovid) Search Strategy 4

CENTRAL (Cochrane Central Register of Controlled Trials) Search Strategy 8

Medline (PubMed) Search Strategy
(“radiomic*”[tw] OR "radiomic signature*"[tw] OR "radiomic feature*"[tw] OR "texture analy*"[tw] OR "quantitative imag*"[tw] OR "image-based biomarker*"[tw] OR "radiogenomic*"[tw] OR "image analy*"[tw] OR "feature extraction"[tw] OR "machine learning"[tw] OR "deep learning"[tw] OR “artificial intelligence”[tw] OR ultrasomic*[tw] OR dosiomic*[tw] AND "Algorithms"[MeSH] OR algorithm*[tw] OR predict*[tw] OR forecast*[tw] OR anticipate*[tw] OR "risk stratification"[tw] OR "prognostic model*"[tw] OR "risk model*"[tw] OR "AI-based prediction"[tw] AND "Cardiac Imaging Techniques"[MeSH] OR “Prognosis” [MeSH] OR detect*[tw] OR diagnos*[tw] OR identif*[tw] OR screen*[tw] OR "image reconstruction"[tw] OR "functional imaging"[tw] OR MRI[tw] OR "cardiac MR"[tw] OR CT[tw] OR "computed tomography"[tw] OR echocardiograph*[tw] OR ultrasound[tw] AND “Cardiovascular diseases”[MeSH] OR “Heart diseases” [MeSH] OR "Cardiomyopathies"[Mesh] OR "Myocarditis"[Mesh] OR “Cardiovascular toxic*”[tw] OR "myocardial dysfunction"[tw] OR "subclinical cardiac dysfunction"[tw] OR "LVEF decline"[tw] OR "left ventricular ejection fraction"[tw] OR LVEF[tw] OR "global longitudinal strain"[tw] OR GLS[tw] OR "cancer therapy-related cardiac dysfunction"[tw] OR "cardiac dysfunction"[tw] OR cardiotoxic*[tw] OR "cardiac tox*"[tw] OR CTRCD[tw] OR "cardiac monitor*"[tw] OR troponin[tw] OR "natriuretic peptide*"[tw] OR BNP[tw] OR NT-proBNP[tw] AND chemotherapy[tw] OR cancer[tw] OR tumor*[tw] OR "cardio-oncolog*"[tw] OR "targeted therapy"[tw] OR immunotherapy[tw] OR "radiation-induced cardiotoxicity"[tw]) OR irradiation[tw] OR "radiation therap*"[tw] OR malignan*[tw] OR neoplasm*[tw])

Scopus Search Strategy
((TITLE-ABS-KEY (artificial intelligence)) OR (TITLE-ABS-KEY (deep learning)) OR (TITLE-ABS-KEY (machine learning)) OR (TITLE-ABS-KEY (image analy*)) OR (TITLE-ABS-KEY (radiogenomic*)) OR (TITLE-ABS-KEY (image-based biomarker*)) OR (TITLE-ABS-KEY (quantitative imag*)) OR (TITLE-ABS-KEY (texture analy*)) OR (TITLE-ABS-KEY (radiomic feature*)) OR (TITLE-ABS-KEY (radiomic signature*)) OR (TITLE-ABS-KEY (radiomic*))) AND (((TITLE-ABS-KEY (AI-based prediction)) OR (TITLE-ABS-KEY (risk model*)) OR (TITLE-ABS-KEY (prognostic model*)) OR (TITLE-ABS-KEY (risk stratification)) OR (TITLE-ABS-KEY (Algorithms)) OR (TITLE-ABS-KEY (algorithm*)) OR (TITLE-ABS-KEY (predict*)) OR (TITLE-ABS-KEY (forecast*)) OR (TITLE-ABS-KEY (anticipate*))) AND (((TITLE-ABS-KEY (chemotherapy)) OR (TITLE-ABS-KEY (cancer)) OR (TITLE-ABS-KEY (tumor*)) OR (TITLE-ABS-KEY (cardio-oncolog*)) OR (TITLE-ABS-KEY (targeted therapy)) OR (TITLE-ABS-KEY (immunotherapy)) OR (TITLE-ABS-KEY (radiation-induced cardiotoxicity)) OR (TITLE-ABS-KEY (irradiation)) OR (TITLE-ABS-KEY (radiation therap*)) OR (TITLE-ABS-KEY (malignan*)) OR (TITLE-ABS-KEY (neoplasm*))) AND ((TITLE-ABS-KEY (Heart diseases)) OR (TITLE-ABS-KEY (Cardiovascular diseases)) OR (TITLE-ABS-KEY (Heart diseases)) OR (TITLE-ABS-KEY (Cardiomyopathies)) OR (TITLE-ABS-KEY (Myocarditis)) OR (TITLE-ABS-KEY (Cardiovascular toxic*)) OR (TITLE-ABS-KEY (myocardial dysfunction)) OR (TITLE-ABS-KEY (subclinical cardiac dysfunction)) OR (TITLE-ABS-KEY (LVEF decline)) OR (TITLE-ABS-KEY (left ventricular ejection fraction)) OR (TITLE-ABS-KEY (LVEF)) OR (TITLE-ABS-KEY (global longitudinal strain)) OR (TITLE-ABS-KEY (GLS)) OR (TITLE-ABS-KEY (cancer therapy-related cardiac dysfunction)) OR (TITLE-ABS-KEY (cardiac monitor*)) OR (TITLE-ABS-KEY (CTRCD)) OR (TITLE-ABS-KEY (cardiac tox*)) OR (TITLE-ABS-KEY (cardiotoxic*)) OR (TITLE-ABS-KEY (cardiac dysfunction)) OR (TITLE-ABS-KEY (troponin)) OR (TITLE-ABS-KEY (natriuretic peptide*)) OR (TITLE-ABS-KEY (BNP)) OR (TITLE-ABS-KEY (NT-proBNP))) AND ((TITLE-ABS-KEY (Cardiac Imaging Techniques)) OR (TITLE-ABS-KEY (Prognosis)) OR (TITLE-ABS-KEY (detect*)) OR (TITLE-ABS-KEY (diagnos*)) OR (TITLE-ABS-KEY (identif*)) OR (TITLE-ABS-KEY ("purchase intencion" and "scale")) OR (TITLE-ABS-KEY (functional imaging)) OR (TITLE-ABS-KEY (image reconstruction)) OR (TITLE-ABS-KEY (screen*)) OR (TITLE-ABS-KEY (ultrasound)) OR (TITLE-ABS-KEY (echocardiograph*)) OR (TITLE-ABS-KEY (computed tomography)) OR (TITLE-ABS-KEY (CT)) OR (TITLE-ABS-KEY (cardiac MR)))))) AND (LIMIT-TO (OA , "all"))

Embase (Ovid) Search Strategy	
#	**Query**
1	Radiomics.mp. [mp=title, abstract, heading word, drug trade name, original title, device manufacturer, drug manufacturer, device trade name, keyword heading word, floating subheading word, candidate term word]
2	radiomic*.mp. [mp=title, abstract, heading word, drug trade name, original title, device manufacturer, drug manufacturer, device trade name, keyword heading word, floating subheading word, candidate term word]
3	1 or 2
4	Forecasting.mp. [mp=title, abstract, heading word, drug trade name, original title, device manufacturer, drug manufacturer, device trade name, keyword heading word, floating subheading word, candidate term word]
5	Prognosis.mp. [mp=title, abstract, heading word, drug trade name, original title, device manufacturer, drug manufacturer, device trade name, keyword heading word, floating subheading word, candidate term word]
6	Clinical Decision Rules.mp. [mp=title, abstract, heading word, drug trade name, original title, device manufacturer, drug manufacturer, device trade name, keyword heading word, floating subheading word, candidate term word]
7	Prediction Methods, Machine.mp. [mp=title, abstract, heading word, drug trade name, original title, device manufacturer, drug manufacturer, device trade name, keyword heading word, floating subheading word, candidate term word]
8	Prediction Algorithms.mp. [mp=title, abstract, heading word, drug trade name, original title, device manufacturer, drug manufacturer, device trade name, keyword heading word, floating subheading word, candidate term word]
9	"Predictive Value of Tests".mp. [mp=title, abstract, heading word, drug trade name, original title, device manufacturer, drug manufacturer, device trade name, keyword heading word, floating subheading word, candidate term word]
10	Risk Assessment.mp. [mp=title, abstract, heading word, drug trade name, original title, device manufacturer, drug manufacturer, device trade name, keyword heading word, floating subheading word, candidate term word]
11	Risk Factors.mp. [mp=title, abstract, heading word, drug trade name, original title, device manufacturer, drug manufacturer, device trade name, keyword heading word, floating subheading word, candidate term word]
12	predict*.mp. [mp=title, abstract, heading word, drug trade name, original title, device manufacturer, drug manufacturer, device trade name, keyword heading word, floating subheading word, candidate term word]
13	foresee.mp. [mp=title, abstract, heading word, drug trade name, original title, device manufacturer, drug manufacturer, device trade name, keyword heading word, floating subheading word, candidate term word]
14	forecast*.mp. [mp=title, abstract, heading word, drug trade name, original title, device manufacturer, drug manufacturer, device trade name, keyword heading word, floating subheading word, candidate term word]
15	anticipate*.mp. [mp=title, abstract, heading word, drug trade name, original title, device manufacturer, drug manufacturer, device trade name, keyword heading word, floating subheading word, candidate term word]
16	4 or 5 or 6 or 7 or 8 or 9 or 10 or 11 or 12 or 13 or 14 or 15

17	Tomography, X-Ray Computed.mp. [mp=title, abstract, heading word, drug trade name, original title, device manufacturer, drug manufacturer, device trade name, keyword heading word, floating subheading word, candidate term word]
18	Ultrasonics.mp. [mp=title, abstract, heading word, drug trade name, original title, device manufacturer, drug manufacturer, device trade name, keyword heading word, floating subheading word, candidate term word]
19	Ultrasonography.mp. [mp=title, abstract, heading word, drug trade name, original title, device manufacturer, drug manufacturer, device trade name, keyword heading word, floating subheading word, candidate term word]
20	Diagnostic Imaging.mp. [mp=title, abstract, heading word, drug trade name, original title, device manufacturer, drug manufacturer, device trade name, keyword heading word, floating subheading word, candidate term word]
21	Magnetic Resonance Imaging.mp. [mp=title, abstract, heading word, drug trade name, original title, device manufacturer, drug manufacturer, device trade name, keyword heading word, floating subheading word, candidate term word]
22	Echocardiography.mp. [mp=title, abstract, heading word, drug trade name, original title, device manufacturer, drug manufacturer, device trade name, keyword heading word, floating subheading word, candidate term word]
23	Detection Algorithms.mp. [mp=title, abstract, heading word, drug trade name, original title, device manufacturer, drug manufacturer, device trade name, keyword heading word, floating subheading word, candidate term word]
24	Early Diagnosis.mp. [mp=title, abstract, heading word, drug trade name, original title, device manufacturer, drug manufacturer, device trade name, keyword heading word, floating subheading word, candidate term word]
25	detect*.mp. [mp=title, abstract, heading word, drug trade name, original title, device manufacturer, drug manufacturer, device trade name, keyword heading word, floating subheading word, candidate term word]
26	distinguish*.mp. [mp=title, abstract, heading word, drug trade name, original title, device manufacturer, drug manufacturer, device trade name, keyword heading word, floating subheading word, candidate term word]
27	identif*.mp. [mp=title, abstract, heading word, drug trade name, original title, device manufacturer, drug manufacturer, device trade name, keyword heading word, floating subheading word, candidate term word]
28	observ*.mp. [mp=title, abstract, heading word, drug trade name, original title, device manufacturer, drug manufacturer, device trade name, keyword heading word, floating subheading word, candidate term word]
29	screen*.mp. [mp=title, abstract, heading word, drug trade name, original title, device manufacturer, drug manufacturer, device trade name, keyword heading word, floating subheading word, candidate term word]
30	diagnos*.mp. [mp=title, abstract, heading word, drug trade name, original title, device manufacturer, drug manufacturer, device trade name, keyword heading word, floating subheading word, candidate term word]
31	imag*.mp. [mp=title, abstract, heading word, drug trade name, original title, device manufacturer, drug manufacturer, device trade name, keyword heading word, floating subheading word, candidate term word]
32	17 or 18 or 19 or 20 or 21 or 22 or 23 or 24 or 25 or 26 or 27 or 28 or 29 or 30 or 31

33	Cardiotoxicity.mp. [mp=title, abstract, heading word, drug trade name, original title, device manufacturer, drug manufacturer, device trade name, keyword heading word, floating subheading word, candidate term word]
34	Ventricular Dysfunction.mp. [mp=title, abstract, heading word, drug trade name, original title, device manufacturer, drug manufacturer, device trade name, keyword heading word, floating subheading word, candidate term word]
35	Heart Failure.mp. [mp=title, abstract, heading word, drug trade name, original title, device manufacturer, drug manufacturer, device trade name, keyword heading word, floating subheading word, candidate term word]
36	cardiac dysfunction.mp. [mp=title, abstract, heading word, drug trade name, original title, device manufacturer, drug manufacturer, device trade name, keyword heading word, floating subheading word, candidate term word]
37	Heart.mp. [mp=title, abstract, heading word, drug trade name, original title, device manufacturer, drug manufacturer, device trade name, keyword heading word, floating subheading word, candidate term word]
38	cardiac monitor*.mp. [mp=title, abstract, heading word, drug trade name, original title, device manufacturer, drug manufacturer, device trade name, keyword heading word, floating subheading word, candidate term word]
39	CTRCD.mp. [mp=title, abstract, heading word, drug trade name, original title, device manufacturer, drug manufacturer, device trade name, keyword heading word, floating subheading word, candidate term word]
40	cardiac tox*.mp. [mp=title, abstract, heading word, drug trade name, original title, device manufacturer, drug manufacturer, device trade name, keyword heading word, floating subheading word, candidate term word]
41	cardiotoxicity*.mp. [mp=title, abstract, heading word, drug trade name, original title, device manufacturer, drug manufacturer, device trade name, keyword heading word, floating subheading word, candidate term word]
42	myocardial dysfunction.mp. [mp=title, abstract, heading word, drug trade name, original title, device manufacturer, drug manufacturer, device trade name, keyword heading word, floating subheading word, candidate term word]
43	33 or 34 or 35 or 36 or 37 or 38 or 39 or 40 or 41 or 42
44	Medical Oncology.mp. [mp=title, abstract, heading word, drug trade name, original title, device manufacturer, drug manufacturer, device trade name, keyword heading word, floating subheading word, candidate term word]
45	Radiation Oncology.mp. [mp=title, abstract, heading word, drug trade name, original title, device manufacturer, drug manufacturer, device trade name, keyword heading word, floating subheading word, candidate term word]
46	Cardiology.mp. [mp=title, abstract, heading word, drug trade name, original title, device manufacturer, drug manufacturer, device trade name, keyword heading word, floating subheading word, candidate term word]
47	Neoplasms.mp. [mp=title, abstract, heading word, drug trade name, original title, device manufacturer, drug manufacturer, device trade name, keyword heading word, floating subheading word, candidate term word]
48	Cardio-Oncology.mp. [mp=title, abstract, heading word, drug trade name, original title, device manufacturer, drug manufacturer, device trade name, keyword heading word, floating subheading word, candidate term word]

49	Radiotherapy.mp. [mp=title, abstract, heading word, drug trade name, original title, device manufacturer, drug manufacturer, device trade name, keyword heading word, floating subheading word, candidate term word]
50	Radiotherapy, Computer-Assisted.mp. [mp=title, abstract, heading word, drug trade name, original title, device manufacturer, drug manufacturer, device trade name, keyword heading word, floating subheading word, candidate term word]
51	Radiation Injuries.mp. [mp=title, abstract, heading word, drug trade name, original title, device manufacturer, drug manufacturer, device trade name, keyword heading word, floating subheading word, candidate term word]
52	Antineoplastic Agents.mp. [mp=title, abstract, heading word, drug trade name, original title, device manufacturer, drug manufacturer, device trade name, keyword heading word, floating subheading word, candidate term word]
53	Immunotherapy.mp. [mp=title, abstract, heading word, drug trade name, original title, device manufacturer, drug manufacturer, device trade name, keyword heading word, floating subheading word, candidate term word]
54	chemotherapy.mp. [mp=title, abstract, heading word, drug trade name, original title, device manufacturer, drug manufacturer, device trade name, keyword heading word, floating subheading word, candidate term word]
55	cancer.mp. [mp=title, abstract, heading word, drug trade name, original title, device manufacturer, drug manufacturer, device trade name, keyword heading word, floating subheading word, candidate term word]
56	cardio-oncolog*.mp. [mp=title, abstract, heading word, drug trade name, original title, device manufacturer, drug manufacturer, device trade name, keyword heading word, floating subheading word, candidate term word]
57	tumor*.mp. [mp=title, abstract, heading word, drug trade name, original title, device manufacturer, drug manufacturer, device trade name, keyword heading word, floating subheading word, candidate term word]
58	44 or 45 or 46 or 47 or 48 or 49 or 50 or 51 or 52 or 53 or 54 or 55 or 56 or 57
59	3 and 16 and 32 and 43 and 58

CENTRAL (Cochrane Central Register of Controlled Trials) Search Strategy	
#	**Query**
1	radiomics:ti,ab,kw
2	radiomic*:ti,ab,kw
3	"radiomic signature*":ti,ab,kw
4	"radiomic feature*":ti,ab,kw
5	"texture analy*":ti,ab,kw
6	"quantitative imag*":ti,ab,kw
7	"image-based biomarker*":ti,ab,kw
8	radiogenomic*:ti,ab,kw
9	"image analy*":ti,ab,kw
10	"feature extraction":ti,ab,kw
11	"machine learning":ti,ab,kw
12	"deep learning":ti,ab,kw
13	"artificial intelligence":ti,ab,kw
14	ultrasomic*:ti,ab,kw
15	dosiomic*:ti,ab,kw
16	#1 OR #2 OR #3 OR #4 OR #5 OR #6 OR #7 OR #8 OR #9 OR #10 OR #11 OR #12 OR #13 OR #14 OR #15
17	MeSH descriptor: [Algorithms] explode all trees
18	algorithm*:ti,ab,kw
19	predict*:ti,ab,kw
20	forecast*:ti,ab,kw
21	anticipate*:ti,ab,kw
22	"risk stratification":ti,ab,kw
23	"prognostic model*":ti,ab,kw
24	"risk model*":ti,ab,kw
25	"AI-based prediction":ti,ab,kw
26	MeSH descriptor: [Prognosis] explode all trees
27	MeSH descriptor: [Risk Assessment] explode all trees
28	#17 OR #18 OR #19 OR #20 OR #21 OR #22 OR #23 OR #24 OR #25 OR #26 OR #27
29	MeSH descriptor: [Cardiac Imaging Techniques] explode all trees
30	MeSH descriptor: [Tomography, X-Ray Computed] explode all trees
31	MeSH descriptor: [Ultrasonography] explode all trees
32	MeSH descriptor: [Echocardiography] explode all trees
33	MeSH descriptor: [Magnetic Resonance Imaging] explode all trees
34	MeSH descriptor: [Positron Emission Tomography Computed Tomography] explode all trees
35	detect*:ti,ab,kw
36	diagnos*:ti,ab,kw
37	identif*:ti,ab,kw
38	screen*:ti,ab,kw
39	"functional imaging":ti,ab,kw
40	"image reconstruction":ti,ab,kw
41	echocardiograph*:ti,ab,kw
42	"computed tomography":ti,ab,kw

43	CT:ti,ab,kw
44	"cardiac MR*":ti,ab,kw
45	MRI:ti,ab,kw
46	ultrasound:ti,ab,kw
47	imag*:ti,ab,kw
48	#29 OR #30 OR #31 OR #32 OR #33 OR #34 OR #35 OR #36 OR #37 OR #38 OR #39 OR #40 OR #41 OR #42 OR #43 OR #44 OR #45 OR #46 OR #47
49	MeSH descriptor: [Cardiovascular Diseases] explode all trees
50	MeSH descriptor: [Heart Diseases] explode all trees
51	MeSH descriptor: [Cardiomyopathies] explode all trees
52	MeSH descriptor: [Myocarditis] explode all trees
53	MeSH descriptor: [Heart Failure] explode all trees
54	MeSH descriptor: [Ventricular Dysfunction] explode all trees
55	"cardiovascular toxic*":ti,ab,kw
56	"myocardial dysfunction":ti,ab,kw
57	"subclinical cardiac dysfunction":ti,ab,kw
58	"LVEF decline":ti,ab,kw
59	"left ventricular ejection fraction":ti,ab,kw
60	LVEF:ti,ab,kw
61	"global longitudinal strain":ti,ab,kw
62	GLS:ti,ab,kw
63	"cancer therapy-related cardiac dysfunction":ti,ab,kw
64	"cardiac dysfunction":ti,ab,kw
65	cardiotoxic*:ti,ab,kw
66	"cardiac tox*":ti,ab,kw
67	CTRCD:ti,ab,kw
68	"cardiac monitor*":ti,ab,kw
69	troponin:ti,ab,kw
70	"natriuretic peptide*":ti,ab,kw
71	BNP:ti,ab,kw
72	NT-proBNP:ti,ab,kw
73	#49 OR #50 OR #51 OR #52 OR #53 OR #54 OR #55 OR #56 OR #57 OR #58 OR #59 OR #60 OR #61 OR #62 OR #63 OR #64 OR #65 OR #66 OR #67 OR #68 OR #69 OR #70 OR #71 OR #72
74	MeSH descriptor: [Neoplasms] explode all trees
75	MeSH descriptor: [Antineoplastic Agents] explode all trees
76	MeSH descriptor: [Radiotherapy] explode all trees
77	MeSH descriptor: [Immunotherapy] explode all trees
78	MeSH descriptor: [Radiation Injuries] explode all trees
79	chemotherapy:ti,ab,kw
80	cancer:ti,ab,kw
81	tumor*:ti,ab,kw
82	"cardio-oncolog*":ti,ab,kw
83	"targeted therapy":ti,ab,kw
84	immunotherapy:ti,ab,kw
85	"radiation-induced cardiotoxicity":ti,ab,kw
86	irradiation:ti,ab,kw
87	"radiation therap*":ti,ab,kw

88	malignan*:ti,ab,kw
89	neoplasm*:ti,ab,kw
90	#74 OR #75 OR #76 OR #77 OR #78 OR #79 OR #80 OR #81 OR #82 OR #83 OR #84 OR #85 OR #86 OR #87 OR #88 OR #89
91	#16 AND #28 AND #48 AND #73 AND #90
